# Physical capacity, occupational physical demands, and relative physical strain of older employees in construction and healthcare

**DOI:** 10.1007/s00420-018-1377-5

**Published:** 2018-11-15

**Authors:** Suzanne L. Merkus, Lars-Kristian Lunde, Markus Koch, Morten Wærsted, Stein Knardahl, Kaj Bo Veiersted

**Affiliations:** National Institute of Occupational Health, Pb 5330 Majorstuen, 0304 Oslo, Norway

**Keywords:** Aging, Muscle strength, Aerobic capacity, Electromyography, Electrocardiography, Inclinometry

## Abstract

**Purpose:**

To assess age-related differences in physical capacity, occupational physical demands, and relative physical strain at a group level, and the balance between capacity and demands at an individual level, for construction and healthcare workers.

**Methods:**

Shoulder strength, back strength, and aerobic capacity were assessed among construction (*n* = 62) and healthcare workers (*n* = 64). During a full working day, accelerometers estimated upper-arm elevation, trunk flexion, and occupational physical activity as indicators of occupational physical demands. Simultaneously, normalised surface electromyography (%sEMG_max_) of the upper trapezius and erector spinae muscles, and normalised electrocardiography (percentage heart rate reserve (%HRR)) estimated relative physical strain. Differences between younger (≤ 44 years) and older (≥ 45 years) workers, as well as the moderating effect of age on the associations between capacity and demands, were analysed per sector.

**Results:**

Compared to younger workers, older workers had similar strength and lower aerobic capacity; older construction workers had similar demands while older healthcare workers had higher demands. Compared to younger workers, older employees had unfavourable muscle activity patterns; %HRR had a tendency to be lower for older construction workers and higher for older healthcare workers. Among construction workers, age moderated the associations between shoulder strength and arm elevation (*p* = 0.021), and between aerobic capacity and occupational physical activity (*p* = 0.040). Age did not moderate these associations among healthcare workers.

**Conclusions:**

In both sectors, the level of occupational physical demands and the higher relative physical strain in older employees require addressing to promote sustainable work participation among an aging population.

**Electronic supplementary material:**

The online version of this article (10.1007/s00420-018-1377-5) contains supplementary material, which is available to authorized users.

## Introduction

Employees in physically demanding occupations, such as in construction and healthcare, are at increased risk for long-term sick leave, disability pensioning, and early retirement (Labriola et al. 2009; Sundstrup et al. 2018). The physical demands of construction and healthcare work, together with the high prevalence rates of musculoskeletal disorders in these sectors (Davis and Kotowski 2015; Umer et al. 2017), are factors that influence the ability to sustain work until retirement age (Jarvholm et al. 2014; Jebens et al. 2014; Jensen et al. 2012; Oude Hengel et al. 2012). As retirement age increases in many countries, insight into determinants of sustainable employability gain importance.

Sustaining employment in physically demanding occupations is largely determined by the balance between physical capacity and occupational physical demands (de Zwart et al. 1995). As a natural process of aging, physical capacity declines with age (Hamberg-van Reenen et al. 2009). This leads to an age-related imbalance between capacity and demands if the level of occupational physical demands is maintained with age. In this situation, older employees will work closer to their maximum capacity and relative physical strain will be higher (de Zwart et al. 1995; Holtermann et al. 2018). Therefore, several studies suggest that lowering physical demands for older employees is crucial to sustaining employment (Jarvholm et al. 2014; Jebens et al. 2014).

Conceptually, age-related changes in the balance between capacity and demands, and the effects on relative strain are straightforward. However, some inconsistencies in the literature regarding comparisons between older and younger employees in physically demanding occupations suggest that the relationships are not so clear-cut. First, although physical capacity declines as a natural process of aging (Hamberg-van Reenen et al. 2009; Soer et al. 2012), some studies among older employees in physically demanding occupations have found that capacity is maintained with age (Gall and Parkhouse 2004; Schibye et al. 2001; Torgen et al. 1999). Second, the recommendation of lowering occupational physical demands with age is not always followed (Aittomaki et al. 2005; Burr et al. 2017; Jarvholm et al. 2014; Jebens et al. 2014; Tonnon et al. 2017). Third, both decreases and increases in relative physical strain with age have been reported (Brighenti-Zogg et al. 2016; Gupta et al. 2014; Jebens et al. 2015).

These inconsistencies may, in part, be attributed to the use of different assessment methods, including subjective assessments and observation (Aittomaki et al. 2005; Burr et al. 2017; Rasmussen et al. 2015; Torgen et al. 1999). These methods are characterised by recall and (self-)estimation bias; for valid assessments, objective measures are preferred (Koch et al. 2016). Additionally, comparisons between older and younger workers at the group level does not provide insight into age-related differences in the balance between physical capacity and occupational physical demands at an individual level. Insight into valid levels of physical capacity, occupational physical demands, and relative physical strain among older employees at a group level, as well as insight into the balance between capacity and demands at an individual level, will improve recommendations and interventions that promote sustainable work participation.

Therefore, using objective assessments, we asked the following research questions for construction and healthcare workers: (1) are there differences between older and younger employees in physical capacity, occupational physical demands, and relative physical strain? (2) Does the association (i.e., the balance) between physical capacity and occupational physical demands depend on age?

## Methods

### Participants and procedures

This cross-sectional study is part of a previously described larger prospective cohort study (Koch et al. 2016; Lunde et al. 2014). From a sample of 594 construction and healthcare workers (response rate 51%) who filled in a questionnaire at baseline, 371 workers consented to participate in objective assessments. From this sample, 66 construction and 72 healthcare workers were selected based on logistics (availability, age, and occupational titles). Exclusion criteria were a diagnosis of cardiovascular disease or pregnancy. All participants underwent a physical examination by a physician or nurse a few days prior to the start of the objective assessments. Simultaneous recordings of occupational physical demands and relative physical strain were done during a full 8 h working day.

### Assessment methods

A comprehensive summary of the methods is provided below; a more in-depth description of the methods is provided in the Supplementary file.

#### Physical capacity

Indicators of physical capacity were physical strength and aerobic capacity. Physical strength was assessed as isometric shoulder strength (kg), tested in a standardized seated position, and isometric back extension strength (kg), tested using a modified Biering-Sørensen test (Bieringsorensen 1984). For each test, the highest value of three attempts was used as maximal voluntary contraction (MVC). A submaximal test on a cycle ergometer estimated aerobic capacity (*V*O_2max_ in L/kg/min) (Astrand 2003). For further information see the Supplementary file and Lunde et al. (2014).

#### Occupational physical demands

Indicators of occupational physical demands were upper-arm elevation, trunk flexion, and occupational physical activity (OPA). Accelerometers were used to estimate these indicators. The duration (percentage of the working day) and frequency (average number per hour) of upper-arm elevations and trunk flexions > 30° and > 60° were calculated (Coenen et al. 2016). OPA was calculated as the duration (percentage of the working day) spent standing, moving, and walking (Skotte et al. 2014). See also the Supplementary file and Lunde et al. (2014).

#### Relative physical strain

Indicators of relative physical strain were the percentage of the normalised surface electromyography (%sEMG_max_) and the percentage of the heart rate reserve (%HRR). Surface electromyography (sEMG) recorded muscle activity of the dominant shoulder (upper trapezius muscle) and of the lower back (both erector spinae longissimus muscles). Recordings were processed and normalised to the maximal muscle activity (sEMG_max_) assessed during the strength tests (Mathiassen et al. 1995). The percentages of the working day with muscle activity > 15% sEMG_max_ (high strain) (Anton et al. 2003; Mathiassen and Winkel 1991) and < 0.5% sEMG_max_ (muscular rest) (Veiersted et al. 1993) were calculated. Additional indicators were the median load level (50th percentile of the amplitude probability distribution function (APDF)); the peak load level (90th percentile APDF); and the frequency (average number per hour) with very high strain activity > 63% sEMG_max_ (Mathiassen and Winkel 1991).

Electrocardiography monitored heart rate and %HRR was calculated using the following equation ($\% {\text{HRR}}=\frac{{{\text{HR}} - {\text{H}}{{\text{R}}_{{\text{min}}}}}}{{{\text{H}}{{\text{R}}_{{\text{max}}}} - {\text{H}}{{\text{R}}_{{\text{min}}}}}} \times 100\%$). Maximal heart rate (HR_max_) was estimated by $208 - (0.7 \times {\text{age}})$ (Tanaka et al. 2001). Minimum heart rate (HR_min_) was defined as the minimum value of a running average of 10 beats during the waking period of all 3–4 assessment days. Average %HRR and the percentage of the working day > 33% HRR (high strain) were calculated (Gupta et al. 2014). For more information see the Supplementary file and Lunde et al. (2014).

#### Demographic, health-related, and work-related information

Participants filled in a questionnaire on demographic, health-related, and work-related information. Based on previous research among aging employees, age was categorised into ≤ 44 and ≥ 45 years (Burr et al. 2017; Schibye et al. 2001; Soer et al. 2012). The questionnaire further included gender, body mass index (BMI), and general health status (SF-36 single-item) (Ware 2000). Pain intensity was assessed for the dominant shoulder and lower back (Kuorinka et al. 1987). Leisure time physical activity and the pursuit of exercises to prevent or treat complaints of the musculoskeletal system were each assessed with a single question. Work-related information included weekly working hours, subjective work ability (Work Ability Index single-item) (Ahlstrom et al. 2010), and subjectively experienced physical work heaviness. Occupational title was categorised according to the level of physical demands into sedentary/light work, medium intensity work, and heavy work as suggested by the Dictionary of Occupational Titles (DOT) (U.S. Department of Labor 1991) and confirmed via visual inspection of our objective assessments. Subscales of the General Questionnaire for Psychological and Social Factors at Work (QPS_Nordic_) assessed job demands (including questions on work pace, overtime, and the amount of work) and control at work (Dallner et al. 2000). A single item from the QPS_Nordic_ assessed whether employees noticed inequalities in how older and younger employees are treated at the workplace. For more detail, see the Supplementary file.

### Data analysis

Analyses were conducted in IBM SPSS (25.0). Statistical significance was set at *p* < 0.05. Due to differences in occupational physical demands between the construction and healthcare sectors, all analyses were stratified for sector. Differences between the age groups (i.e., ≤ 44 years and ≥ 45 years) for demographic, health-related, and subjective work-related variables, as well as for physical capacity, occupational physical demands, and relative physical strain indicators were tested with Fisher’s exact tests for categorical variables, *t* tests for normally distributed continuous variables, and Mann–Whitney *U* tests for skewed continuous variables. Sensitivity analyses assessed age-related differences in physical capacity, occupational physical demands, and relative physical strain for employees at increased risk of early exit from work, i.e., for employees with occupations that are medium and heavy according to DOT.

Regression analyses studied whether the association between occupational physical demands and physical capacity depended on age, i.e., whether age had a moderating effect on the association. Non-normally distributed dependent variables were transformed. Dependent variables were duration of arm elevation > 60° (natural logarithm transformation), duration of trunk flexion > 60°, and duration standing/moving/walking (squared transformation). Regression coefficients for arm elevation were back transformed (*e*^*b*^ = Exp(*b*)), as were coefficients for standing/moving/walking (square root(*b*) = Sqrt(*b*)). For each dependent variable, the respective capacity indicator, i.e., shoulder strength, back strength, and aerobic capacity, were added as independent variables, together with age (≤ 44 and ≥ 45), and the interaction between age and the capacity indicator. Additional analyses identified explanatory variables of interaction effects. Potential variables were chosen based on logic and prior research: gender, general health, pain, preventative exercises, leisure time physical activity, psychosocial work factors, work ability, and level of physical demands (DOT). A variable was considered to be explanatory when an interaction changed by > 10% after adding the variable to the model.

## Results

From the 138 selected participants, 12 employees were unable to participate due to various practical reasons, leading to a final study sample of 126 employees (construction *n* = 62; healthcare *n* = 64). The construction participants included project managers, supervisors, engineers, bricklayers, carpenters, concrete workers, and assistants. The healthcare participants included managers, nurses, nursing assistants, social educators, and kitchen and cleaning staff. Data from accelerometers were available from 92 to 95% of the participants (arm elevation *n* = 120; trunk flexion *n* = 115; activities *n* = 119). sEMG data for the upper trapezius muscle were available from 75% of the participants (construction *n* = 39; healthcare *n* = 56), and for the erector spinae muscles from 40% of the participants (construction *n* = 22; healthcare *n* = 28). Heart rate data were available from 81% of the participants (construction *n* = 46; healthcare *n* = 56). From the questionnaire, nine out of the 14 questions used in this study missed 2–19% of the answers; the remaining questions were complete.

The average age for construction workers was 39.9 years (SD 13.4 years; range 19–67 years), and for healthcare workers 44.1 years (SD 9.9 years; range 20–64 years). The sample of construction workers consisted of 61 males and one female, while one-fifth of the healthcare workers were male (Table 1). Both the construction and healthcare samples included occupations with sedentary/light physical demands according to the DOT classification; however, medium intensity work was not represented among construction workers, and heavy intensity work was not represented among healthcare workers. There was a tendency that older construction workers more often had sedentary/light physical work. Compared to their younger colleagues, older healthcare workers were more likely to perceive their work as moderate to somewhat heavy (*p* = 0.013). In both sectors, younger workers reported that older and younger workers were not treated equally; the difference between the age groups reached statistical significance for construction workers (*p* < 0.001) (Table 1).

**Table 1:** Demographic, health-related, and subjective work-related information for older (≥ 45 years) and younger (≤ 44 years) employees in construction and healthcare

	Construction				Healthcare			
	Total *n* = 62	≤ 44 years *n* = 39	≥ 45 years *n* = 23	*p*	Total *n* = 64	≤ 44 years *n* = 33	≥ 45 years *n* = 31	*p*
	*n* (%)	*n* (%)	*n* (%)		*n* (%)	*n* (%)	*n* (%)	
Gender								
Male	61 (98)	38 (97)	23 (100)	0.629	14 (22)	8 (24)	6 (19)	0.337
Female	1 (2)	1 (3)	0 (0)		50 (78)	25 (76)	25 (81)	
Level of physical demands (DOT)								
Sedentary/light work	16 (26)	8 (21)	8 (35)	0.242	8 (13)	5 (15)	3 (10)	0.709
Medium intensity work	–	–	–		56 (87)	28 (85)	28 (90)	
Heavy work	46 (74)	31 (79)	15 (65)		–	–	–	
General health								
Very good to excellent	28 (45)	21 (54)	7 (30)	0.192	30 (47)	14 (42)	16 (52)	0.568
Good	26 (42)	14 (36)	12 (52)		25 (39)	13 (40)	12 (38)	
Poor to fair	8 (13)	4 (10)	4 (18)		9 (14)	6 (18)	3 (10)	
BMI								
Normal (< 25 kg/m^2^)	25 (40)	17 (44)	8 (35)	0.596	34 (53)	20 (61)	14 (45)	0.316
Overweight (> 25 kg/m^2^)	37 (60)	22 (56)	15 (65)		30 (47)	13 (39)	17 (55)	
Shoulder pain								
No pain—a little pain	50 (85)	32 (87)	18 (82)	0.715	40 (67)	16 (53)	24 (80)	0.054
Moderate—severe pain	9 (15)	5 (13)	4 (18)		20 (33)	14 (47)	6 (20)	
Lower back pain								
No pain—a little pain	45 (74)	30 (79)	15 (65)	0.368	40 (65)	18 (56)	22 (73)	0.192
Moderate—severe pain	16 (26)	8 (21)	8 (35)		22 (35)	14 (44)	8 (27)	
Subjective work demands								
Not at all—light	15 (24)	8 (21)	7 (30)	0.177	21 (33)	**14 (44)**	**7 (23)**	**0.013**
Moderate—somewhat heavy	31 (50)	23 (58)	8 (35)		27 (43)	**8 (25)**	**19 (61)**	
Heavy—very, very heavy+	15 (26)	8 (21)	8 (35)		15 (24)	**10 (31)**	**5 (16)**	
Preventative exercises								
No	21 (34)	12 (31)	9 (39)	0.583	13 (20)	7 (21)	6 (19)	1.000
Yes	41 (66)	27 (69)	14 (61)		51 (80)	26 (79)	25 (81)	
Leisure physical activity								
Sedentary—some activity	40 (71)	22 (67)	18 (78)	0.385	38 (73)	16 (64)	22 (82)	0.215
Regular—hard activity	16 (29)	11 (33)	5 (22)		14 (27)	9 (36)	5 (18)	

	Construction				Healthcare			
	Total *n* = 62	≤ 44 years *n* = 39	≥ 45 years *n* = 23	*p*	Total *n* = 64	≤ 44 years *n* = 33	≥ 45 years *n* = 31	*p*
	*m* (SD)	*m* (SD)	*m* (SD)		*m* (SD)	*m* (SD)	*m* (SD)	
Age (years)	39.9 (13.4)	**31.0 (6.5)**	**55.1 (6.7)**	< **0.001**	44.1 (9.9)	**36.3 (6.0)**	**52.5 (5.1)**	< **0.001**
Weekly working hours	37.8 (4.0)	37.6 (5.0)	38.2 (1.1)	0.542	35.7 (4.2)	35.7 (3.3)	35.6 (5.0)	0.919
Work ability (0–10)	8.9 (1.5)	8.5 (1.7)	9.4 (0.8)	0.054	8.8 (1.3)	8.7 (1.2)	8.9 (1.5)	0.398
QPS job demands (1–5)	3.0 (0.6)	3.1 (0.5)	2.9 (0.6)	0.276	3.0 (0.7)	3.0 (0.8)	3.0 (0.7)	0.814
QPS job control (1–5)	3.2 (0.7)	3.2 (0.7)	3.3 (0.8)	0.483	2.9 (0.8)	3.0 (0.8)	2.8 (0.7)	0.454
Age inequality (1–5)	2.1 (0.9)	**2.4 (0.8)**	**1.6 (0.7)**	< **0.001**	2.1 (1.2)	2.4 (1.4)	1.8 (0.9)	0.088

Bold typeface indicates statistically significant differences between age groups at *p* < 0.05

### Physical capacity

Due to the strong dependency on sex, physical strength and aerobic capacity are reported separately for male and female workers (Table 2). Construction workers ≥ 45 years had lower aerobic capacity than male construction workers ≤ 44 years (*p* = 0.001); shoulder and back strength were similar between the age groups. Compared to their younger colleagues, male and female healthcare workers ≥ 45 years had lower aerobic capacity; however, the differences did not reach statistical significance.

**Table 2:** Physical capacity estimated by shoulder strength, back strength, and aerobic capacity for older (≥ 45 years) and younger (≤ 44 years) construction and healthcare workers, stratified by gender

	Shoulder strength (kg)			Back strength (kg)			*V*O_2max_ (L/min/kg)		
	*n*	*m* (SD)	*p*	*n*	*m* (SD)	*p*	*n*	*m* (SD)	*p*
Construction									
Male (*n* = 61)									
≤ 44 years	38	28.2 (6.9)		38	48.4 (14.8)		34	**41.3 (12.2)**	
≥ 45 years	23	28.2 (9.7)	0.990	21	49.0 (17.5)	0.879	22	**33.1 (5.2)**	**0.001**
Healthcare									
Male (*n* = 14)									
≤ 44 years	8	24.2 (5.2)		8	47.1 (23.0)		7	38.7 (13.1)	
≥ 45 years	6	24.4 (6.9)	0.953	6	38.6 (13.8)	0.440	6	26.7 (7.2)	0.071
Female (*n* = 50)									
≤ 44 years	25	15.4 (3.6)		24	30.2 (10.2)		24	33.4 (9.0)	
≥ 45 years	25	13.6 (4.4)	0.108	25	26.9 (10.7)	0.275	23	29.0 (5.1)	0.091

Bold typeface indicates statistically significant differences between age groups at *p* < 0.05

### Occupational physical demands

For construction workers, no statistically significant age differences were found for duration or frequency of arm elevation and trunk flexion > 30° and > 60°, nor for duration of OPA, although older workers had a tendency for shorter duration of trunk flexion > 60° than younger workers (Table 3).

**Table 3:** Occupational physical demands estimated by upper-arm elevation, trunk flexion, and occupational physical activity (OPA) for older (≥ 45 years) and younger (≤ 44 years) construction and healthcare workers

	Age (year)	Construction					Healthcare				
		*n* (%)	Median	*m* (SD)	Min–max	*p*	*n* (%)	Median	*m* (SD)	Min–max	*p*
Upper-arm elevation											
> 30° duration (% working day)	≤ 44	37 (100)	41.3	41.9 (11.6)	22.4–82.1	0.907	30 (100)	30.0	29.8 (12.5)	10.0–56.4	0.291
	≥ 45	22 (100)	41.8	41.5 (16.3)	5.3–79.5		31 (100)	32.3	32.8 (8.7)	11.7–48.2	
> 30° frequency (#/h)	≤ 44	37 (100)	37.0	38.1 (14.0)	15.6–62.5	0.900	30 (100)	**27.6**	**28.2 (9.1)**	**10.7–48.3**	**0.017**
	≥ 45	22 (100)	37.6	36.4 (17.7)	1.7–62.0		31 (100)	**35.1**	**35.9 (13.6)**	**15.9–81.1**	
> 60° duration (% working day)	≤ 44	37 (100)	7.6	8.6 (4.9)	1.1–17.6	0.888	30 (100)	**2.9**	**3.2 (1.7)**	**1.3–8.5**	**0.039**
	≥ 45	22 (100)	8.0	8.7 (6.1)	0.5–21.0		31 (100)	**3.9**	**4.3 (2.3)**	**0.8–11.0**	
> 60° frequency (#/h)	≤ 44	37 (100)	8.7	9.8 (6.2)	1.5–23.6	0.638	30 (100)	**3.6**	**4.1 (1.9)**	**1.5–8.7**	**0.045**
	≥ 45	22 (100)	6.5	9.0 (6.7)	0.1–22.3		31 (100)	**5.3**	**5.5 (2.7)**	**1.3–10.8**	
> 60° for > 10% working day (% working day)	≤ 44	15 (41)	13.1	13.8 (1.9)	10.8–17.6	0.466	0 (0)				
	≥ 45	8 (36)	14.6	15.3 (83.6)	11.0–21.0		1 (3)				
Trunk flexion											
> 30° duration (% working day)	≤ 44	33 (100)	18.6	20.6 (10.2)	2.7–50.8	0.314	30 (100)	**16.4**	**17.5 (8.1)**	**4.3–35.7**	**0.002**
	≥ 45	22 (100)	16.1	17.9 (9.0)	8.0–42.5		30 (100)	**25.0**	**24.4 (8.4)**	**3.2–44.8**	
> 30° frequency (#/h)	≤ 44	33 (100)	21.0	24.2 (13.6)	3.3–65.2	0.236	30 (100)	**17.6**	**20.5 (8.9)**	**4.8–41.2**	**0.001**
	≥ 45	22 (100)	17.2	20.2 (11.3)	7.0–46.7		30 (100)	**27.7**	**30.9 (14.2)**	**5.0–71.3**	
> 60° duration (% working day)	≤ 44	33 (100)	9.0	8.7 (6.1)	0.3–29.1	0.086	30 (100)	**3.5**	**4.7 (3.3)**	**0.7–12.8**	**0.008**
	≥ 45	22 (100)	3.9	6.5 (6.5)	0.7–26.7		30 (100)	**7.4**	**7.5 (4.3)**	**0.1–19.2**	
> 60 frequency (#/h)	≤ 44	33 (100)	9.2	10.4 (8.1)	0.5–41.5	0.118	30 (100)	**4.2**	**5.3 (3.6)**	**1.1–14.5**	**0.002**
	≥ 45	22 (100)	5.5	7.4 (6.0)	0.7–20.4		30 (100)	**9.3**	**9.9 (6.2)**	**0.3–24.7**	
> 60° for > 10% working day (% working day)	≤ 44	15 (45)	10.9	13.6 (5.2)	10.1–29.1	0.349	2 (7)	11.9	11.9 (1.3)	11.0–12.8	
	≥ 45	5 (23)	16.1	16.3 (5.9)	10.2–26.7		7 (23)	12.2	13.5 (2.8)	11.3–19.2	
Occupational physical activity											
Stand/move/walk (% working day)	≤ 44	37 (100)	76.5	71.1 (18.9)	18.4–92.5	0.962	30 (100)	62.1	60.3 (22.9)	13.1–93.4	0.657
	≥ 45	22 (100)	80.4	63.6 (28.9)	9.4–93.0		30 (100)	67.1	63.9 (18.5)	23.6–91.5	
Stand/move/walk > 75% working day (% working day)	≤ 44	22 (59)	80.2	82.0 (5.6)	75.2–92.5	0.200	11 (37)	84.6	84.1 (4.9)	75.5–93.4	0.657
	≥ 45	13 (59)	83.8	84.1 (4.6)	76.1–93.0		10 (33)	80.7	81.7 (5.2)	75.9–91.5	

Bold typeface indicates statistically significant differences between age groups at *p* < 0.05

With exception of duration of arm elevation > 30°, older healthcare workers (≥ 45 years) had statistically significantly higher duration and frequency of arm elevation and trunk flexion > 30° and > 60° than younger healthcare workers (≤ 44 years) (Table 3). There was no statistically significant difference between the age groups for OPA (Table 3).

### Relative physical strain

Older construction workers had statistically significantly longer duration of upper trapezius muscle activity > 15% sEMG_max_, shorter duration of muscle rest, higher median and peak load levels, and a higher frequency of activity > 63% sEMG_max_ than their younger colleagues (Table 4). A similar increased tendency of erector spinae muscle activity was seen amongst construction workers ≥ 45 years; however, differences between the age groups were not statistically significant (Table 4). Compared to younger construction workers, workers ≥ 45 years had a lower average %HRR and spent less time > 33%HRR; however, differences did not reach statistical significance (Table 4).

**Table 4:** Relative physical strain estimated by muscle activity of the upper trapezius and erector spinae muscles (%sEMG_max_) and %HRR for older (≥ 45 years) and younger (≤ 44 years) construction and healthcare workers

	Age (year)	Construction					Healthcare				
		*n* (%)	Median	*m* (SD)	Min–max	*p*	*n* (%)	Median	*m* (SD)	Min–max	*p*
Upper trapezius muscle activity											
> 15% sEMG_max_ (% working day)	≤ 44	21 (100)	**3.9**	**5.4 (4.2)**	**0.7–13.2**	**0.004**	27 (100)	**3.4**	**6.0 (5.4)**	**0.8–17.2**	**0.018**
	≥ 45	18 (100)	**8.3**	**12.7 (10.1)**	**1.4–36.1**		29 (100)	**11.3**	**11.9 (10.0)**	**0.9–32.3**	
Muscular rest (% working day)	≤ 44	21 (100)	**17.8**	**21.7 (12.4)**	**2.3–55.5**	**0.003**	27 (100)	9.5	12.7 (11.6)	1.1–39.1	0.594
	≥ 45	18 (100)	**9.6**	**12.3 (9.3)**	**2.9–40.9**		29 (100)	11.5	12.2 (7.8)	0.4–29.4	
Median load (%sEMG_max_)	≤ 44	21 (100)	**2.0**	**2.3 (1.3)**	**0.4–4.9**	**0.005**	27 (100)	2.8	3.6 (2.3)	0.8–9.3	0.166
	≥ 45	18 (100)	**3.9**	**4.5 (2.7)**	**1.2–10.0**		29 (100)	3.5	4.4 (2.6)	1.0–9.6	
Peak load (%sEMG_max_)	≤ 44	21 (100)	**10.1**	**10.6 (4.1)**	**4.9–18.0**	**0.004**	27 (100)	**10.0**	**11.0 (4.3)**	**5.8–17.8**	**0.019**
	≥ 45	18 (100)	**13.8**	**16.8 (8.2)**	**7.8–40.0**		29 (100)	**15.9**	**15.4 (7.1)**	**5.7–33.1**	
Freq. > 63% sEMG_max_ (#/h)	≤ 44	19 (90)	**3.8**	**6.4 (6.5)**	**0.3–20.8**	**0.033**	25 (93)	**1.5**	**3.6 (4.5)**	**0.2–14.8**	**0.014**
	≥ 45	17 (94)	**11.1**	**36.2 (72.1)**	**0.1–302.4**		29 (100)	**4.9**	**13.7 (26.7)**	**0.3–121.7**	
Erector spinae muscle activity											
> 15% sEMG_max_ (% working day)	≤ 44	14 (100)	7.5	9.5 (7.5)	1.6–24.5	0.212	15 (100)	**6.5**	**10.4 (11.0)**	**0.4–37.3**	**0.019**
	≥ 45	8 (100)	14.3	20.3 (17.8)	1.3–53.4		13 (100)	**19.6**	**22.9 (14.1)**	**2.4–44.8**	
Muscular rest (% working day)	≤ 44	14 (100)	9.0	14.2 (13.0)	2.7–47.8	0.110	15 (100)	**12.4**	**16.0 (13.7)**	**1.6–47.4**	**0.025**
	≥ 45	8 (100)	2.7	8.3 (11.0)	0.1–30.5		13 (100)	**4.1**	**5.9 (5.0)**	**1.2–16.6**	
Median load (%sEMG_max_)	≤ 44	14 (100)	3.5	3.5 (1.8)	0.6–5.7	0.238	15 (100)	**3.1**	**3.6 (2.9)**	**0.6–10.4**	**0.007**
	≥ 45	8 (100)	6.4	6.6 (5.2)	1.1–16.3		13 (100)	**5.9**	**7.2 (3.4)**	**2.7–12.5**	
Peak load (%sEMG_max_)	≤ 44	14 (100)	13.2	14.2 (4.9)	8.1–23.6	0.212	15 (100)	**12.5**	**14.1 (8.4)**	**4.2–33.1**	**0.013**
	≥ 45	8 (100)	17.6	20.3 (11.6)	7.0–44.7		13 (100)	**20.5**	**22.9 (9.7)**	**8.0–42.1**	
Freq. > 63% sEMG_max_ (#/h)	≤ 44	11 (79)	1.9	8.2 (13.5)	0.1–45.8	0.525	11 (73)	1.7	11.7 (25.1)	0.2–84.0	0.051
	≥ 45	6 (75)	5.2	54.6 (123.3)	1.2–306.2		12 (92)	6.2	66.7 (74.9)	0.9–254.0	
%HRR											
Average %HRR	≤ 44	29 (100)	34.0	33.2 (10.4)	15.2–54.5	0.097	30 (100)	23.9	25.5 (5.7)	17.9–36.8	0.079
	≥ 45	17 (100)	28.5	29.2 (5.6)	21.8–44.3		26 (100)	30.1	29.1 (8.6)	13.7–45.7	
> 33% HRR (% working day)	≤ 44	29 (100)	55.2	49.5 (34.1)	1.8–93.5	0.054	30 (100)	12.0	21.4 (21.2)	0.4–64.7	0.071
	≥ 45	17 (100)	26.2	31.1 (18.9)	6.8–75.0		26 (100)	35.5	35.4 (26.8)	0.04–84.6	

Bold typeface indicates statistically significant differences between age groups at *p* < 0.05

Older healthcare workers had statistically significantly longer duration of upper trapezius muscle activity > 15% sEMG_max_, as well as a higher peak load level and frequency of activity > 63% sEMG_max_ than their younger colleagues (Table 4). All parameters of the erector spinae muscles were statistically significantly higher for older compared to younger healthcare workers (Table 4). Increases in average %HRR and duration > 33%HRR for healthcare workers ≥ 45 years compared to those ≤ 44 years were not statistically significant (Table 4).

### Sensitivity analyses

The sensitivity analyses that included medium and heavy occupations only, resulted in two deviations from the analyses involving the whole sample (See Tables 1, 2, 3 in the Supplementary file). First, average %HRR (*p* = 0.021) and duration > 33% HRR (*p* = 0.009) were statistically significantly lower for older than younger constructions workers. Second, arm elevation > 60° regarding duration (*p* = 0.066) and frequency (*p* = 0.081) were no longer statistically significantly different for older and younger healthcare workers.

### Association between capacity and demands in relation to age

**Fig. 1:**
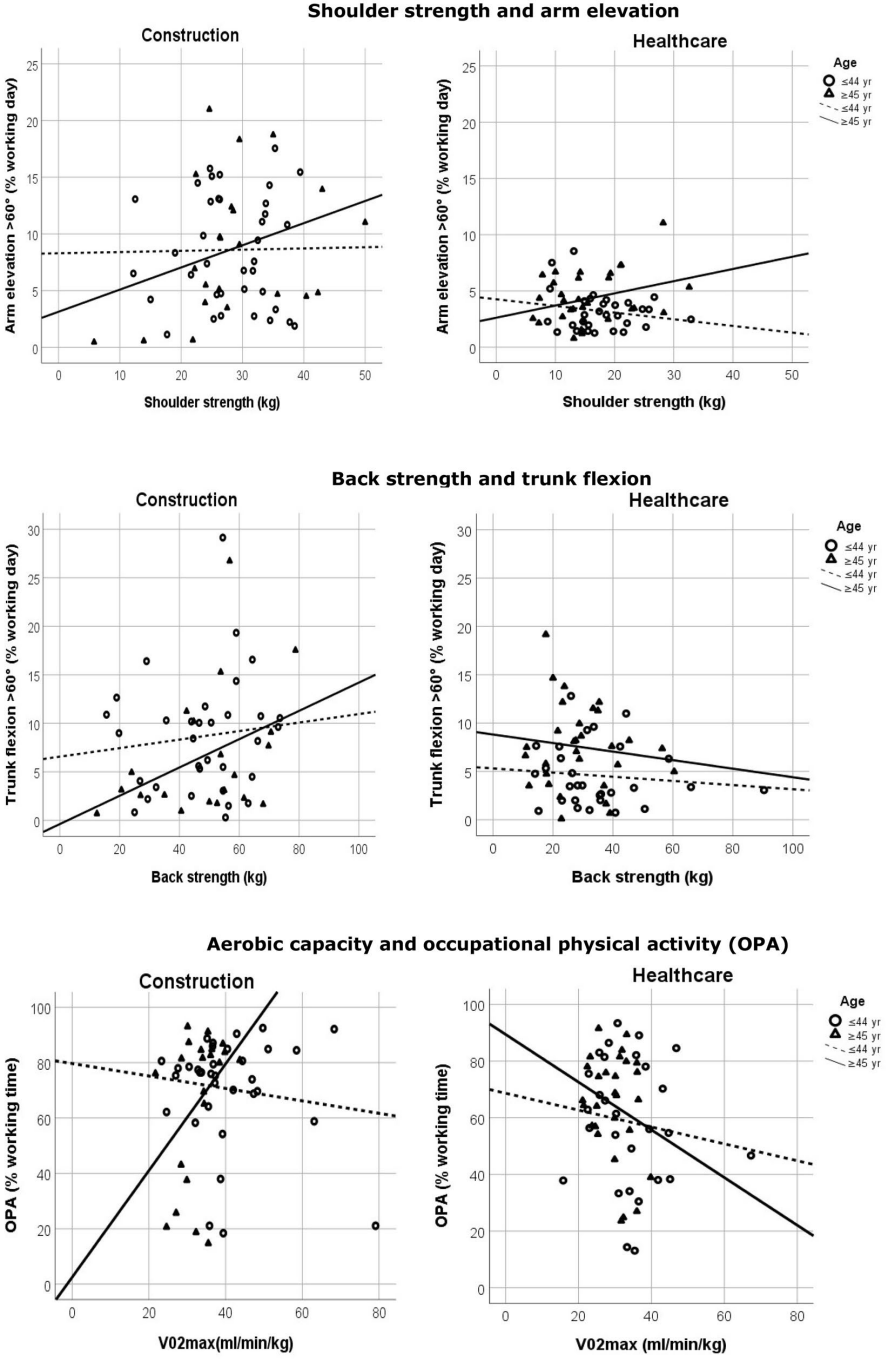
The age-dependent associations between shoulder strength and arm elevation > 60°, between back strength and trunk flexion > 60°, and between occupational physical activity and aerobic capacity (*V*O_2max_) among construction workers (left column) and healthcare workers (right column)

Among construction workers, age modified the associations between shoulder strength and duration of arm elevation > 60° (shoulder strength × age *b* = 1.07; *p* = 0.021), and between aerobic capacity and OPA (aerobic capacity × age *b* = 15.21; *p* = 0.040) (Table 5). The positive interaction effects indicated that construction employees ≥ 45 years were more likely to have a better balance between arm elevation > 60° and shoulder strength, and between OPA and aerobic capacity, than employees ≤ 44 years (Fig. 1). In the analyses that identified explanatory variables of the interaction effects, the largest change—and thereby best explanatory effect—was obtained by adding the categorical level of physical work demands (DOT) to the models. After adding the categorical level of physical work demands (DOT) to the models, the interactions were no longer statistically significant (shoulder strength × age *p* = 0.218; aerobic capacity × age *p* = 0.734) (Table 5). The association between back strength and the duration of trunk flexion > 60° was not modified by age in construction workers (back strength × age *p* = 0.338) (Table 5). For healthcare workers, age did not modify the associations between shoulder strength and duration of arm elevation > 60° (shoulder strength × age *p* = 0.214); between back strength and duration of trunk flexion > 60° (back strength × age *p* = 0.760); nor between aerobic capacity and OPA (aerobic capacity × age *p* = 0.686) (Table 5; Fig. 1).

**Table 5:** Age modification of the associations between arm elevation > 60° and shoulder strength, between trunk flexion > 60° and back strength, and between occupational physical activity and *V*O_2max_, respectively

Construction (*n* = 62)	Arm elevation > 60° (% working day)^a^	Trunk flexion > 60° (% working day)^b^	Occupational physical activity (% working day)^c^
	Exp(*b*) (95% CI)	*b* (95% CI)	Sqrt(*b*) (95% CI)
	*n* = 59	*n* = 53	*n* = 55
Crude model			
Capacity indicator	1.00 (0.96–1.04)	0.04 (− 0.10 to 0.18)	− 3.66 (− 9.19 to 7.60)
Age			
≤ 44 years	1.00	0.00	0.00
≥ 45 years	0.14 (0.03–0.68)	− 6.72 (− 17.60 to 4.15)	− 89.59 (− 124.96 to 20.95)
Capacity indicator × age	**1.07 (1.01–1.12)**	0.11 (− 1.00 to 0.33)	**15.21 (3.38–21.24)**
Adjusted model			
Capacity indicator	1.01 (0.98–1.04)		5.83 (− 1.97 to 8.47)
Age			
≤ 44 years	1.00		0.00
≥ 45 years	0.45 (0.13–1.60)		− 14.54 (− 66.13 to 62.85)
Capacity indicator × age	1.03 (0.98–1.07)		4.52 (− 9.97 to 11.84)
Level of work demands			
Sedentary/light work	1.00		0.00
Heavy work	**3.43 (2.30–5.11)**		**69.69 (63.48–75.39)**

Healthcare (*n* = 64)	Arm elevation > 60° (% working day)^a^	Trunk flexion > 60° (% working day)^b^	Occupational physical activity (% working day)^c^
	Exp(*b*) (95% CI)	*b* (95% CI)	Sqrt(*b*) (95% CI)
	*n* = 61	*n* = 59	*n* = 56
Crude model			
Capacity indicator	0.99 (0.96–1.03)	− 0.02 (− 0.11 to 0.07)	− 5.92 (− 11.23 to 7.48)
Age			
≤ 44 years	1.00	0.00	0.00
≥ 45 years	0.81 (0.34–1.92)	3.54 (− 1.52 to 8.59)	40.34 (− 68.06 to 88.81)
Capacity indicator × age	1.03 (0.98–1.08)	− 0.03 (− 0.18 to 0.12)	− 6.38 (− 15.54 to 12.65)

Bold typeface indicates statistically significant differences between age groups at p < 0.05

^a^Capacity indicator for arm elevation > 60° is shoulder strength (kg)

^b^Capacity indicator for trunk flexion > 60° is back strength (kg)

^c^Capacity indicator for occupational physical activity is aerobic capacity (*V*O_2max_)

## Discussion

This study assessed age-related differences in physical capacity, occupational physical demands, and relative physical strain at a group level, as well as age-related differences in the balance between capacity and demands at an individual level among construction and healthcare workers. Compared to their younger colleagues, older construction and healthcare workers had similar physical strength yet poorer aerobic capacity. Older workers had similar or higher occupational physical demands compared to younger workers. Relative physical strain, estimated by the pattern of relative muscle activity in the shoulder and lower back, was unfavourable for older workers. Relative physical strain, estimated by percentage of the heart rate reserve, had a tendency to be lower for older construction workers, specifically for those with heavy physical demands, and had a tendency to be higher for older healthcare workers. At an individual level, older employees in construction were more likely to have a better balance between capacity and demands compared to their younger colleagues. This was explained by the higher percentage of older participants having sedentary/light work. For healthcare workers, the balance between capacity and demands at an individual level was similar for both age groups.

Studies of the general population (Soer et al. 2012) and of various physically demanding occupations (Nygard et al. 1991) suggest that both strength and aerobic capacity decline with age. Contrastingly, our study showed that only aerobic capacity was lower among older workers, while strength was similar for older and younger workers. These findings in our sample of construction and healthcare workers are consistent with findings among male power line technicians (Gall and Parkhouse 2004) and waste collectors (Schibye et al. 2001). This may suggest that some physically demanding work may contribute to maintaining strength, but not aerobic capacity (Gall and Parkhouse 2004; Jebens et al. 2015; Schibye et al. 2001; Soer et al. 2012; Torgen et al. 1999).

The similar or higher levels of occupational physical demands among the older construction and healthcare workers in our sample is not in line with recommendations to reduce demands as employees age (Aittomaki et al. 2005; Burr et al. 2017; Jarvholm et al. 2014; Jebens et al. 2014). This is of concern, because the level of occupational physical demands measured in our sample have previously been associated with musculoskeletal disorders (Coenen et al. 2016) and occupational physical demands may have stronger negative health effects for older employees than for younger employees (Burr et al. 2017). Therefore, the level of occupational physical demands among older construction and healthcare workers requires attention to preserve health and promote sustainable work participation until retirement age (Jebens et al. 2014; Oude Hengel et al. 2012).

Age-related differences in relative physical strain depended on the sector. Among healthcare workers, older employees worked at higher levels of relative strain; this is in line with their higher self-reported exertion levels. Among construction workers, older employees, specifically those with heavier work demands, worked at a lower percentage of their heart rate reserve yet with an unfavourable muscle activity pattern. This paradox may suggest that older construction workers had strategies to reduce some, but not all heavier physical demands. Such strategies may include delegating heavier tasks to younger workers, as suggested by the tendency for older workers to have less trunk flexion and the unequal treatment reported by younger construction workers. Nevertheless, the unfavourable muscle activity pattern seen among older employees in both sectors, suggests that their tasks were more strenuous for them than the tasks of the younger workers. Maintaining muscular strength with age may not be sufficient to protect against a potential detrimental muscle activity pattern; therefore, physical demands may need to be reduced for older employees to reduce the risk for musculoskeletal disorders.

At a group level, our findings suggest that the older workers in our study may have had a poorer balance between capacity and demands than the younger workers, e.g., older workers had lower aerobic capacity yet similar occupational physical activity compared to their younger colleagues. This is in line with previous hypotheses and expectations (de Zwart et al. 1995; Holtermann et al. 2018). However, at an individual level, our findings show a different association. At an individual level, the balance was likely better for older than younger construction workers, while it is similar for older and younger healthcare workers. Among the construction employees, this indicates that older workers with lower capacity had lower demands, while those with higher capacity had higher demands. In comparison, younger construction workers seemed to have similar demands, irrespective of capacity (Fig. 1). Older and younger healthcare workers too, seemed to have similar demands irrespective of capacity (Fig. 1).

The ‘better’ balance for older construction workers and ‘similar’ balance for older healthcare workers in our sample may be interpreted in a number of ways. First, the fact that the level of physical work demands (DOT) explained the better balance for construction workers, may suggest that the overrepresentation of older construction workers in our sample with sedentary/light work (who also had lower capacity) is due to a possibility to move into sedentary/light work (e.g., supervisors or project leaders) with age. Moving into lighter work may be a way to meet previously reported needs to reduce physical demands to remain at work (Jebens et al. 2014). The similar balance for older and younger healthcare workers suggests that they may not have such opportunities. Second, a better and similar balance—rather than a poorer balance—may have been observed owing to a healthy worker survival effect. Potentially, the older employees were a self-selected sample who remained at work, because they had maintained their muscle strength and work ability. Further, the better or similar balance of older employees is compared to the balance of younger employees whose balance may not have been optimal: in both sectors younger workers seemed to have similar demands irrespective of capacity. Therefore, the comparison with younger workers did not provide insight into whether a ‘better’ or ‘similar’ balance is a ‘good’ balance or sufficient to maintain health and sustain work. All three interpretations argue for reducing occupational physical demands with age as a means to promote sustainable work participation.

### Strengths and limitations

A major strength of this study is the use of objective measurements that eliminated recall and self-estimation bias. In addition, the simultaneous assessments of several indicators of occupational physical demands and relative physical strain provided a comprehensive description of the effects of physically demanding occupations on aging employees. A limitation of this study was its cross-sectional design that did not follow the development of age-related changes over time. This may have given rise to a healthy worker survival effect among older employees that may have led to an overestimation of physical capacity and an underestimation of relative physical strain. However, this only supports the need to address the level of occupational physical demands to avoid premature dropout from the labour market. Furthermore, the sample size was relatively small and not randomly selected; therefore, the findings need to be interpreted with caution. However, a small sample size enabled several simultaneous objective measurements of occupational exposures and a close follow-up of each participant throughout their working day. Another limitation is the loss of data, specifically for the lower back sEMG assessments owing to electrodes falling off during a warm, sweaty summer. This likely led to power problems in the statistical analysis among construction workers; however, the substantial differences between the age groups may still be related to an increased risk for fatigue and musculoskeletal disorders.

### Future research

The high level of occupational physical demands and relative physical strain among older employees in our sample urges future research to identify ways in which work can be organised that reduces physical demands and relative strain among older employees. In doing so, it should be recognised that similar exposure limits may have stronger effects on health among older than younger employees (Burr et al. 2017); therefore, dose–response relationships in relation to age should be identified. When assessing capacity, future studies may consider using dynamic capacity test, such as used in functional capacity assessments, in addition to the isometric capacity tests used in the present study. Where isometric tests determine the individual’s general physical capacity, dynamic functional capacity tests reflect specific task demands more precisely. Moreover, future studies are suggested to assess physical capacity, occupational physical demands, and relative physical strain repeatedly over time to gain insight into how these variables change in the same individuals over time.

## Concluding remarks

At a group level, when compared to younger workers, older employees had similar physical strength, yet lower aerobic capacity; however, the occupational physical demands were similar or higher. The muscle activity pattern was unfavourable for older healthcare workers, and percentage heart rate reserve had a tendency to be higher. For older construction workers, the muscle activity pattern was unfavourable, while percentage heart rate reserve had a tendency to be lower. At an individual level, the better balance between capacity and demands for older construction workers suggests that they may have opportunities to cope with the heavy physical demands. The similar balance for older and younger healthcare workers suggests that older healthcare workers may not have such opportunities. Overall, the level of occupational demands and relative physical strain, and the balance between capacity and demands among older employees, argue for reducing occupational physical demands with age as an incentive to promote sustainable work participation.

## Electronic supplementary material

Below is the link to the electronic supplementary material.

Supplementary material 1 (DOCX 45 KB)
