# Supplementary material for: Physical capacity, occupational physical demands, and relative physical strain of older employees in construction and healthcare
Source: Int Arch Occup Environ Health. 2018 Nov 15;92(3):295–307. doi: 10.1007/s00420-018-1377-5 (PMC6420471; doi:10.1007/s00420-018-1377-5)
Supplement: Supplementary file 1 — Supplementary material 1 (DOCX 45 KB) [file 420_2018_1377_MOESM1_ESM.docx]

**Supplementary file**

**Physical capacity, occupational physical demands, and relative physical strain of older employees in construction and healthcare**

*Suzanne L. Merkus^1^, Lars-Kristian Lunde^1^, Markus Koch^1^, Morten Wærsted^1^, Stein Knardahl^1^, Kai Bo Veiersted^1^

**^1^** National Institute of Occupational Health, Gydas vei 8, 0336 Oslo, Norway

*Address corresponding author e-mail address: [suzanne.merkus@stami.no](mailto:suzanne.merkus@stami.no)

**EXTENSIVE DESCRIPTION OF THE METHODS**

An extensive description of the assessment methods is provided in this Appendix for the paper “Do occupational physical demands correspond to individual capacity among older construction and healthcare workers such that relative physical strain is reduced?”.

**Assessment methods**

*Physical capacity*

Indicators of physical capacity were isometric shoulder strength, isometric back strength, and aerobic capacity*. Isometric shoulder strength* was tested in a standardized seated position, with the upper arm on the dominant side elevated in 90° abduction in the scapular plane and pulling upwards against a hanging scale (Kern HBC HCN 100K200IP, KERN and SOHN GmbH, Balingen, Germany). The hanging scale was attached proximal to epicondyles of the humerus on the one side (at 90° relative to the upper-arm) and to the floor on the other. *Isometric back extension strength* was tested using a modified Biering-Sørensen test (Bieringsorensen 1984): participants wore a harness on their upper-body while lying face down on a bench with their upper-body suspended, and were urged to pull a wire attached to the floor via a hanging scale. Each test was performed three times with verbal motivation given at each attempt to ensure the participants performed at their maximal capacity. The highest value of the three attempts were used as maximal voluntary contraction (MVC). Strength tests were perform prior to the start of the assessed working day. *Aerobic capacity* (VO_2max_ in L/kg/min) was estimated using a submaximal test on a cycle ergometer (Ergometer 839 E, Varberg, Sweden) (Astrand 2003) according to the Åstrand nomogram (Astrand and Ryhming 1954) and adjusted for age (Astrand 1960). Aerobic capacity tests were conducted during the physical exam a few days prior to the start of the objective assessments. For further information on the test procedures see Lunde et al. (2014).

*Occupational physical demands*

Indicators of occupational physical demands were upper-arm elevation, trunk flexion, and occupational physical activity (OPA). These indicators were estimated using accelerometers (Actigraph GT3X+, Actigraph, Florida, U.S.A) placed on the upper arm, upper back, right hip, and right thigh. Data were gathered for 3-4 consecutive days; however, for this study, data were used from the first working day only. Duration and frequency of upper-arm elevation and trunk flexion (Coenen et al. 2016) were estimated using custom-made software. Upper-arm elevation was calculated as the duration (percentage of the working day) and frequency (number per hour) with the dominant arm elevated >30° and >60° relative to gravity during a full working day. Trunk flexion was calculated as the duration (percentage of the working day) and frequency (number per hour) with upper body bent forward >30° and >60° (with reference in an upraised position) during non-seated activities (Villumsen et al. 2015).

OPA was estimated by the duration spent standing, moving, and walking as a percentage of the full working day (Korshoj et al. 2014; Skotte et al. 2014). The accelerometers placed on the right thigh and hip, or on the right thigh and upper back, identified the activities standing, moving, and walking in the custom-made software package Acti4 (National Research Centre for the Working Environment, Copenhagen, Denmark and Federal Institute of Occupational Safety and Health, Berlin, Germany) (Korshoj et al. 2014; Skotte et al. 2014). For further information see Lunde et al. (2014).

*Relative physical strain*

Indicators of relative physical strain were the percentage of the normalised surface Electromyography (%sEMG_max_) and the percentage of the heart rate reserve (%HRR). Heart rate data were recorded for 3-4 consecutive working days, while surface Electromyography (sEMG) data were gathered for the first working day only. For this study, data from both sEMG and heart rate assessments on the first working day were used.

Muscle activity for the dominant shoulder (Upper trapezius muscle) and bilaterally for the lower back (Erector Spinae longissimus muscle) was recorded by sEMG. Assessments were done using self-adhesive pre-gelled Ag/AgCl electrodes (Ambu Neuroline 720, Ambu, Ballerup, Copenhagen) placed parallel to the underlying muscle fibres in standardised positions (Lunde et al. 2014). Maximum electromyography values (sEMG_max_) for the Upper trapezius and Erector Spinae muscles were determined during the strength tests of the shoulder and back. For further information see Lunde et al. (2014).

The RAW sEMG data were examined for movement artefacts and electromagnetic interference and excluded where appropriate (Hansson et al. 2003). The data were root-mean-squared (RMS) converted and normalised to a percentage of the sEMG_max_. For the Erector Spinae muscles, sEMG recordings from the right side were used and—where not available—substituted by sEMG recordings from the left side. For both the Upper trapezius and Erector Spinae muscles, the duration of relatively high strain was calculated, i.e. percentage of the working day spent >15% sEMG_max_ (Anton et al. 2003; Mathiassen and Winkel 1991), as well as the duration of muscular rest, i.e. percentage of the working day spent <0.5% sEMG_max_ (Nordander et al. 2000; Veiersted et al. 1993). Additionally, median and peak load levels, i.e. 50% and 90% of the amplitude probability distribution function (APDF), respectively, (Jonsson 1982; Veiersted et al. 2013) were calculated, as well as the frequency (number per hour) of muscle activations >63% sEMG_max_ (Mathiassen and Winkel 1991).

Heart rate was monitored with electrocardiography using Actiheart (Camntech, Cambridge, United Kingdom) (Brage et al. 2006). Electrodes were attached at the apex of the sternum and on the left intercostal muscles at the level of the 6^th^ and 7^th^ costae. Analog signals of the Actiheart were filtered (10 Hz–35 Hz). For more information see Lunde et al. (2014) and Lunde et al. (2016). Heart Rate Reserve (HRR) was calculated using the ‘Karvonen method’ with the equation $HRR=\frac{HR-HRmin}{HRmax-HRmin}$. Maximal heart rate (HR_max_) was estimated by $208-(0.7*age)$ (Tanaka et al. 2001). Minimum heart rate (HR_min_) was defined as the minimum value of a running average of 10 beats during the waking period of all 3-4 assessment days. Average %HRR and the percentages of the working day >33% HRR (high strain) were calculated.

*Demographic, health-related, and work-related information*

Participants filled in a questionnaire on demographic, health-related, and work-related information. Demographic information included gender (male/female). Based on previous research among aging employees, age was categorised into ≤44 and ≥45 years (Burr et al. 2017; Schibye et al. 2001; Soer et al. 2012). Health-related information included height and weight to calculate body mass index (BMI). General health status was assessed with a single-item question from the SF-36 with responses from 1 ‘excellent’ to 5 ‘poor’ (Ware 2000). Pain intensity for the neck, shoulder, and lower-back regions was scored a 4-point scale ranging from 0 ‘no pain’ to 3 ‘severe pain’; a mannequin drawing indicated the body regions (Kuorinka et al. 1987). A single question assessed leisure time physical activity the past year; the four answer categories were dichotomised into ‘sedentary or some activity at least 4 hours per week’ and ‘physical exercise to hard physical exercise several times a week’. Whether participants performed exercises or trained to treat or prevent complaints of the back, neck, shoulders, arms, or legs was assessed with a dichotomous answer (no/yes).

The participants were asked to provide work-related information that included weekly working hours. Occupational title was categorised according to the level of physical demands into sedentary/light work, medium intensity work, and heavy work as suggested by the U.S. Department of Labor and confirmed via visual inspection of our data (U.S. Department of Labor 1991). Subjective work ability was assessed with a single-item question from the Work Ability Index on ‘current work ability compared to the lifetime best’ that was answered on an 11-point scale from 0 ‘completely unable to work’ to 10 ‘work ability at its best’ (Ahlstrom et al. 2010; Ilmarinen 2007). Subjectively experienced physical work heaviness was assessed with a single item “*How physically heavy is your work usually?”* that was answered on a 13-point scale from 0 ‘not at all’ to >10 ‘maximally heavy’. The answers were divided into three categories for analyses: 1) not at all to light, 2) moderate to somewhat heavy, and 3) heavy to maximally heavy. Psychosocial factors were assessed using the General Questionnaire for Psychological and Social Factors at Work (QPS_Nordic_) (Dallner et al. 2000). Job demands were assessed with the quantitative demands subscale that included questions on work pace, overtime, and the amount of work. Control at work was assessed with the control of decision subscale, and age inequality was assessed with a single item “*Have you noticed any inequalities in how older and younger employees are treated at your workplace?*”. The psychosocial factors were answered on a 5-point scale from 1 ‘very seldom or never’ to 5 ‘very often or always’ for job demands and control at work, and from 1 ‘very little or not at all’ to 5 ‘very much’ for inequality.

**Data cleaning**

For all objective assessment methods, data from each participant was visually inspected for validity. For example, when an accelerometer fell off, or when activities were invalid (e.g. the suggested activity was rowing) or highly unlikely (e.g. a participant never sat, also not during breaks), the data for that specific assessment were excluded for that individual. Additionally, accelerometer, sEMG, and heart rate data registrations that were shorter than the full working day (arbitrarily chosen as <50% of the working day) were visually inspected for representativeness of the assessment day against the other registered data. For example, when sEMG data was only available for the morning, it was included if the morning activities were representative of the afternoon activities, arm elevation, and heart rate. Further, heart rate assessments were excluded when beat error exceeded 50%, defined as heart rate <35 or >230 bpm or >15% difference between two succeeding beats.

**SENSITIVITY ANALYSES**

**Table 1. Physical capacity estimated by shoulder and back strength, and aerobic capacity for older (≥45 years) and younger (≤44 years) employees without sedentary/light work in construction (heavy work) and healthcare (medium intensity work), stratified by gender.**

|  | **Shoulder strength**  **(kg)** | | | |  | **Back strength**  **(kg)** | | | |  | **VO_2_max**  **(L/min/kg)** | | | |
| --- | --- | --- | --- | --- | --- | --- | --- | --- | --- | --- | --- | --- | --- | --- |
|  | **n** | **m** | **(sd)** | **p** |  | **n** | **m** | **(sd)** | **p** |  | **n** | **m** | **(sd)** | **p** |
| **Construction (heavy work)** | | | | |  |  |  |  |  |  |  |  |  |  |
| ***Male*** *(n=46)* |  |  |  |  |  |  |  |  |  |  |  |  |  |  |
| ≤44 years | 31 | 27.3 | (7.0) |  |  | 31 | 47.8 | (15.6) |  |  | 27 | **39.5** | **(10.3)** |  |
| ≥45 years | 15 | 31.2 | (8.3) | 0.105 |  | 15 | 55.0 | (15.0) | 0.139 |  | 15 | **34.4** | **(5.3)** | **0.041** |
| **Healthcare (medium intensity work)** | | | | |  |  |  |  |  |  |  |  |  |  |
| ***Male*** *(n=13)* |  |  |  |  |  |  |  |  |  |  |  |  |  |  |
| ≤44 years | 7 | 24.6 | (5.5) |  |  | 7 | 49.9 | (23.2) |  |  | 6 | 39.3 | (14.2) |  |
| ≥45 years | 6 | 24.4 | (6.9) | 0.958 |  | 6 | 38.6 | (13.8) | 0.305 |  | 6 | 26.7 | (7.2) | 0.092 |
| ***Female*** *(n=43)* |  |  |  |  |  |  |  |  |  |  |  |  |  |  |
| ≤44 years | 21 | 15.8 | (3.3) |  |  | 20 | 30.8 | (9.9) |  |  | 20 | 32.8 | (9.5) |  |
| ≥45 years | 22 | 13.4 | (4.6) | 0.072 |  | 22 | 27.4 | (11.2) | 0.311 |  | 20 | 29.5 | (5.1) | 0.181 |

*Bold typeface indicates statistically significant differences between age groups at p<0.05*

|  |  | **Construction (heavy work)** | | | | | | |  | **Healthcare (medium intensity work)** | | | | | | |
| --- | --- | --- | --- | --- | --- | --- | --- | --- | --- | --- | --- | --- | --- | --- | --- | --- |
|  | **age** | **n** | **(%)** | **median** | **mean** | **(sd)** | **min** – **max** | **p** |  | **n** | **(%)** | **median** | **mean** | **(sd)** | **min** – **max** | **p** |
| Arm elevation |  |  |  |  |  |  |  |  |  |  |  |  |  |  |  |  |
| >30° duration (% working day) | ≤44 yr | 29 | 100 | 42.2 | 42.6 | (8.8) | 23.3 – 66.0 | 0.839 |  | 25 | (100) | 23.6 | 30.0 | (12.9) | 14.3 – 56.4 | 0.213 |
|  | ≥45 yr | 15 | (100) | 41.9 | 43.1 | (6.6) | 32.9 – 57.3 |  |  | 28 | (100) | 34.3 | 33.8 | (8.1) | 14.6 – 48.2 |  |
| >30° frequency (#/hr) | ≤44 yr | 29 | (100) | 41.9 | 43.2 | (11.3) | 20.0 – 62.5 | 0.683 |  | 25 | (100) | **29.3** | **29.5** | **(8.6)** | **16.8 – 48.3** | **0.023** |
|  | ≥45 yr | 15 | (100) | 45.3 | 44.5 | (12.2) | 26.7 – 62.0 |  |  | 28 | (100) | **35.5** | **37.1** | **(13.3)** | **18.4 – 81.1** |  |
| >60° duration (% working day) | ≤44 yr | 29 | (100) | 10.8 | 10.1 | (4.4) | 2.4 – 17.6 | 0.665 |  | 25 | (100) | 3.4 | 3.5 | (1.7) | 1.3 – 8.5 | 0.066 |
|  | ≥45 yr | 15 | (100) | 11.0 | 11.1 | (5.5) | 3.9 – 21.0 |  |  | 28 | (100) | 4.2 | 4.6 | (2.2) | 1.2 – 11.0 |  |
| >60°frequency (#/hr) | ≤44 yr | 29 | (100) | 11.9 | 11.7 | (5.7) | 1.7 – 23.6 | 0.990 |  | 25 | (100) | 4.4 | 4.5 | (1.9) | 1.9 – 8.7 | 0.081 |
|  | ≥45 yr | 15 | (100) | 11.7 | 11.9 | (6.1) | 4.2 – 22.3 |  |  | 28 | (100) | 5.4 | 5.8 | (2.6) | 1.6 – 10.8 |  |
| >60° for >10% working day  (% working day) | ≤44 yr | 15 | (52) | 13.1 | 13.8 | (1.9) | 10.8 – 17.6 | 0.466 |  | 0 | (0) |  |  |  |  |  |
|  | ≥45 yr | 8 | (53) | 14.6 | 15.3 | (3.6) | 11.0 – 21.0 |  |  | 1 | (4) | 11.0 |  |  |  |  |
| Trunk flexion |  |  |  |  |  |  |  |  |  |  |  |  |  |  |  |  |
| >30° duration (% working day) | ≤44 yr | 25 | (100) | 23.4 | 23.7 | (9.4) | 3.4 – 50.8 | 0.367 |  | 25 | (100) | **16.3** | **17.1** | **(7.7)** | **4.3 – 34.7** | **<0.001** |
|  | ≥45 yr | 14 | (100) | 17.7 | 20.9 | (9.3) | 10.3 – 42.5 |  |  | 27 | (100) | **25.3** | **25.3** | **(7.4)** | **13.2 – 44.8** |  |
| >30° frequency (#/hr) | ≤44 yr | 25 | (100) | 25.6 | 28.6 | (12.4) | 4.3 –65.2 | 0.216 |  | 25 | (100) | **19.0** | **21.5** | **(9.3)** | **4.8 – 41.2** | **0.001** |
|  | ≥45 yr | 14 | (100) | 21.6 | 24.8 | (10.6) | 13.0 – 46.7 |  |  | 27 | (100) | **28.3** | **32.5** | **(13.7)** | **9.9 – 71.3** |  |
| >60° duration (% working day) | ≤44 yr | 25 | (100) | 10.2 | 10.6 | (5.7) | 0.8 – 29.1 | 0.071 |  | 25 | (100) | **3.5** | **4.5** | **(3.1)** | **0.7 – 12.8** | **0.004** |
|  | ≥45 yr | 14 | (100) | 5.8 | 8.2 | (7.3) | 1.7 – 26.7 |  |  | 27 | (100) | **7.5** | **7.8** | **(4.3)** | **0.7 – 19.2** |  |
| >60 frequency (#/hr) | ≤44 yr | 25 | (100) | 10.8 | 12.8 | (7.9) | 1.3 –41.5 | 0.098 |  | 25 | (100) | **4.2** | **5.7** | **(3.7)** | **1.1 – 14.5** | **0.005** |
|  | ≥45 yr | 14 | (100) | 8.1 | 9.3 | (6.0) | 2.3 – 20.4 |  |  | 27 | (100) | **9.4** | **10.3** | **(6.3)** | **0.7 – 24.7** |  |
| >60° for >10% working day  (% working day) | ≤44 yr | 15 | (60) | 10.9 | 13.6 | (5.2) | 10.1 –29.1 | 0.307 |  | 1 | (4) |  | 12.8 |  |  |  |
|  | ≥45 yr | 4 | (29) | 16.4 | 17.4 | (6.9) | 10.2 – 26.7 |  |  | 7 | (26) | 12.2 | 13.5 | (2.8) | 11.3 – 19.2 |  |
| Occupational physical activity |  |  |  |  |  |  |  |  |  |  |  |  |  |  |  |  |
| Stand/move/walk  (% working day) | ≤44 yr | 29 | (100) | 78.5 | 79.0 | (7.6) | 62.2 – 92.5 | 0.055 |  | 25 | (100) | 66.1 | 64.9 | (18.6) | 30.5 – 93.4 | 0.791 |
|  | ≥45 yr | 14 | (100) | 83.2 | 83.1 | (5.9) | 69.4 – 93.0 |  |  | 27 | (100) | 68.8 | 66.7 | (16.2) | 23.6 – 91.5 |  |
| Stand/move/walk 75% working day  (% working day) | ≤44 yr | 22 | (76) | 80.2 | 82.0 | (5.6) | 75.2 – 92.5 | 0.169 |  | 10 | (40) | 83.8 | 83.6 | (4.9) | 75.5 – 93.4 | 0.315 |
|  | ≥45 yr | 13 | (93) | 83.8 | 84.1 | (4.6) | 76.1 –93.0 |  |  | 10 | (37) | 80.7 | 81.7 | (5.2) | 75.9 – 91.5 |  |

**Table 2. Occupational physical demands estimated by arm elevation, trunk flexion, and occupational physical activity (OPA) for older (≥45 years) and younger (≤44 years) employees without sedentary/light work in construction (heavy work) and healthcare (medium intensity work).**

*Bold typeface indicates statistically significant differences between age groups at p<0.05*

**Table 3. Relative physical strain estimated by muscle activity of the Upper trapezius and Erector spinae muscles (%sEMGmax) and %HRR for older (≥45 years) and younger (≤44 years) employees without sedentary/light work in construction (heavy work) and healthcare (medium intensity work).**

|  | **Construction (heavy work)** | | | | | | | |  | **Healthcare (medium work)** | | | | | | |
| --- | --- | --- | --- | --- | --- | --- | --- | --- | --- | --- | --- | --- | --- | --- | --- | --- |
|  | **age** | **n** | **(%)** | **median** | **mean** | **(sd)** | **min** – **max** | **p** |  | **n** | **(%)** | **median** | **mean** | **(sd)** | **min** – **max** | **p** |
| Upper trapezius muscle activity | |  |  |  |  |  |  |  |  |  |  |  |  |  |  |  |
| >15% EMG_max_ (% working day) | ≤44 yr | 16 | (100) | **5.2** | **6.6** | **(4.2)** | **1.1 – 13.2** | **0.005** |  | 22 | (100) | **3.9** | **5.8** | **(5.3)** | **1.0 – 17.2** | **0.006** |
|  | ≥45 yr | 13 | (100) | **14.5** | **15.8** | **(10.3)** | **3.4 – 36.1** |  |  | 26 | (100) | **12.4** | **13.0** | **(10.0)** | **1.4 – 32.3** |  |
| Muscular rest (% working day) | ≤44 yr | 16 | (100) | **17.4** | **17.9** | **(8.7)** | **2.3 – 37.8** | **0.015** |  | 22 | (100) | 9.3 | 12.6 | (11.7) | 1.1 – 39.1 | 0.710 |
|  | ≥45 yr | 13 | (100) | **7.6** | **11.0** | **(7.0)** | **2.9 – 25.9** |  |  | 26 | (100) | 11.4 | 12.0 | (8.2) | 0.4 – 29.4 |  |
| Median load (%EMG_max_) | ≤44 yr | 16 | (100) | **2.1** | **2.6** | **(1.2)** | **0.8 – 4.9** | **0.020** |  | 22 | (100) | 2.6 | 3.5 | (2.3) | 0.8 – 9.3 | 0.094 |
|  | ≥45 yr | 13 | (100) | **3.9** | **4.9** | **(2.9)** | **1.5 – 10.0** |  |  | 26 | (100) | 3.5 | 4.6 | (2.6) | 1.0 – 9.6 |  |
| Peak load (%EMG_max_) | ≤44 yr | 16 | (100) | **10.7** | **11.8** | **(3.9)** | **6.4 – 18.0** | **0.007** |  | 22 | (100) | **9.3** | **10.7** | **(4.3)** | **5.8 – 17.8** | **0.005** |
|  | ≥45 yr | 13 | (100) | **19.1** | **19.0** | **(8.6)** | **7.8 – 40.0** |  |  | 26 | (100) | **16.5** | **16.1** | **(7.0)** | **6.7 –33.1** |  |
| Freq. EMG peaks >63% EMG_max_ (#/hr) | ≤44 yr | 15 | (94) | **4.8** | **8.0** | **(6.5)** | **1.0 – 20.8** | **0.033** |  | 16 | (73) | **1.5** | **3.2** | **(4.1)** | **0.2 – 14.8** | **0.003** |
|  | ≥45 yr | 13 | (100) | **14.6** | **46.0** | **(80.6)** | **1.3 – 302.3** |  |  | 13 | (50) | **6.8** | **15.1** | **(27.9)** | **0.3 – 121.7** |  |
| Erector Spinae muscle activity | |  |  |  |  |  |  |  |  |  |  |  |  |  |  |  |
| >15% EMG_max_ (% working day) | ≤44 yr | 11 | (100) | 11.0 | 10.7 | (8.0) | 1.6 – 24.5 | 0.375 |  | 12 | (100) | **9.3** | **12.2** | **(11.6)** | **0.4 – 37.3** | **0.045** |
|  | ≥45 yr | 7 | (100) | 16.2 | 21.4 | (18.9) | 1.3 – 53.4 |  |  | 12 | (100) | **19.7** | **24.6** | **(13.2)** | **7.7 – 44.8** |  |
| Muscular rest (% working day) | ≤44 yr | 11 | (100) | 7.5 | 10.0 | (6.2) | 2.7 – 23.5 | 0.069 |  | 12 | (100) | 8.5 | 13.8 | (14.0) | 1.6 – 47.4 | 0.101 |
|  | ≥45 yr | 7 | (100) | 2.3 | 5.2 | (6.8) | 0.1 – 18.1 |  |  | 12 | (100) | 3.7 | 5.8 | (5.2) | 1.2 – 16.6 |  |
| Median load (%EMG_max_) | ≤44 yr | 11 | (100) | 4.0 | 3.9 | (1.6) | 2.1 – 5.7 | 0.211 |  | 12 | (100) | **3.7** | **4.1** | **(3.0)** | **0.6 – 10.4** | **0.028** |
|  | ≥45 yr | 7 | (100) | 7.5 | 7.4 | (5.1) | 1.9 – 16.3 |  |  | 12 | (100) | **6.2** | **7.5** | **(3.3)** | **2.7 – 12.5** |  |
| Peak load (%EMG_max_) | ≤44 yr | 11 | (100) | 15.8 | 15.1 | (5.1) | 8.9 – 23.6 | 0.425 |  | 12 | (100) | **14.6** | **15.5** | **(8.7)** | **4.2 – 33.1** | **0.028** |
|  | ≥45 yr | 7 | (100) | 17.5 | 20.7 | (12.4) | 7.0 – 44.7 |  |  | 12 | (100) | **21.3** | **24.1** | **(9.0)** | **13.1 – 42.1** |  |
| Freq. EMG peaks >63% EMG_max_ (#/hr) | ≤44 yr | 8 | (73) | 6.6 | 10.9 | (15.1) | 0.1 – 45.8 | 1.000 |  | 9 | (75) | 1.7 | 14.0 | (27.5) | 0.2 – 84.0 | 0.129 |
|  | ≥45 yr | 5 | (71) | 6.6 | 64.7 | (135.0) | 1.2 – 306.2 |  |  | 12 | (100) | 6.2 | 36.7 | (74.9) | 0.9 – 254.0 |  |
| %HRR |  |  |  |  |  |  |  |  |  |  |  |  |  |  |  |  |
| Average %HRR | ≤44 yr | 22 | (100) | **36.9** | **36.5** | **(8.5)** | **17.2 – 54.5** | **0.021** |  | 26 | (100) | 25.1 | 26.4 | (5.5) | 18.3 – 36.8 | 0.103 |
|  | ≥45 yr | 11 | (100) | **29.4** | **30.2** | **(6.1)** | **23.1 – 44.3** |  |  | 25 | (100) | 30.5 | 29.7 | (8.2) | 13.7 – 45.7 |  |
| >33% HRR (% working day) | ≤44 yr | 22 | (100) | **64.2** | **59.4** | **(25.2)** | **3.4 – 93.5** | **0.009** |  | 26 | (100) | 14.0 | 23.9 | (21.8) | 0.4 – 64.7 | 0.101 |
|  | ≥45 yr | 11 | (100) | **31.3** | **34.8** | **(20.6)** | **6.8 – 75.0** |  |  | 25 | (100) | 38.9 | 36.8 | (26.4) | 0.04 – 84.6 |  |

*Bold typeface indicates statistically significant differences between age groups at p<0.05*

**References**

Ahlstrom L, Grimby-Ekman A, Hagberg M, Dellve L (2010) The work ability index and single-item question: associations with sick leave, symptoms, and health--a prospective study of women on long-term sick leave. Scand J Work Environ Health 36(5):404-12

Anton D, Cook TM, Rosecrance JC, Merlino LA (2003) Method for quantitatively assessing physical risk factors during variable noncyclic work. Scand J Work Environ Health 29(5):354-62

Astrand I (1960) Aerobic work capacity in men and women with special reference to age. Acta Physiol Scand Suppl 49(169):1-92

Astrand P-O (2003) Textbook of work physiology : physiological bases of exercise, 4th edn. Human Kinetics, Champaign, IL

Astrand PO, Ryhming I (1954) A nomogram for calculation of aerobic capacity (physical fitness) from pulse rate during sub-maximal work. J Appl Physiol 7(2):218-21

Bieringsorensen F (1984) Physical Measurements as Risk Indicators for Low-Back Trouble over a One-Year Period. Spine 9(2):106-119 doi:Doi 10.1097/00007632-198403000-00002

Brage S, et al. (2006) Effect of combined movement and heart rate monitor placement on physical activity estimates during treadmill locomotion and free-living. European Journal of Applied Physiology 96(5):517-524 doi:10.1007/s00421-005-0112-6

Burr H, Pohrt A, Rugulies R, Holtermann A, Hasselhorn HM (2017) Does age modify the association between physical work demands and deterioration of self-rated general health? Scand J Work Env Hea 43(3):241-249 doi:10.5271/sjweh.3625

Coenen P, Douwes M, van den Heuvel S, Bosch T (2016) Towards exposure limits for working postures and musculoskeletal symptoms - a prospective cohort study. Ergonomics 59(9):1182-92 doi:10.1080/00140139.2015.1130862

Dallner M, et al. (2000) Validation of the General Nordic Questionnaire (QPSNordic) for Psychological and Social Factors at Work. Nord, Copenhagen (Denmark): Nordic Council of Ministers

Hansson GA, Asterland P, Kellerman M (2003) Modular data logger system for physical workload measurements. Ergonomics 46(4):407-15 doi:10.1080/0014013021000034920

Ilmarinen J (2007) The Work Ability Index (WAI). Occup Med-Oxford 57(2):160-160 doi:10.1093/occmed/kqm008

Jonsson B (1982) Measurement and evaluation of local muscular strain in the shoulder during constrained work. J Hum Ergol (Tokyo) 11(1):73-88

Korshoj M, et al. (2014) Validity of the Acti4 software using ActiGraph GT3X+accelerometer for recording of arm and upper body inclination in simulated work tasks. Ergonomics 57(2):247-53 doi:10.1080/00140139.2013.869358

Kuorinka I, et al. (1987) Standardised Nordic questionnaires for the analysis of musculoskeletal symptoms. Appl Ergon 18(3):233-7

Lunde LK, et al. (2014) Musculoskeletal health and work ability in physically demanding occupations: study protocol for a prospective field study on construction and health care workers. BMC Public Health 14:1075 doi:10.1186/1471-2458-14-1075

Lunde LK, Koch M, Veiersted KB, Moen GH, Waersted M, Knardahl S (2016) Heavy Physical Work: Cardiovascular Load in Male Construction Workers. Int J Environ Res Public Health 13(4):356 doi:10.3390/ijerph13040356

Mathiassen SE, Winkel J (1991) Quantifying variation in physical load using exposure-vs-time data. Ergonomics 34(12):1455-68 doi:10.1080/00140139108964889

Nordander C, et al. (2000) Muscular rest and gap frequency as EMG measures of physical exposure: the impact of work tasks and individual related factors. Ergonomics 43(11):1904-19 doi:10.1080/00140130050174536

Schibye B, Hansen AF, Sogaard K, Christensen H (2001) Aerobic power and muscle strength among young and elderly workers with and without physically demanding work tasks. Appl Ergon 32(5):425-31

Skotte J, Korshoj M, Kristiansen J, Hanisch C, Holtermann A (2014) Detection of physical activity types using triaxial accelerometers. J Phys Act Health 11(1):76-84 doi:10.1123/jpah.2011-0347

Soer R, Brouwer S, Geertzen JH, van der Schans CP, Groothoff JW, Reneman MF (2012) Decline of functional capacity in healthy aging workers. Arch Phys Med Rehabil 93(12):2326-32 doi:10.1016/j.apmr.2012.07.009

Tanaka H, Monahan KD, Seals DR (2001) Age-predicted maximal heart rate revisited. J Am Coll Cardiol 37(1):153-6

U.S. Department of Labor EaTA (1991) Dictionary of Occupational Titles. In: U.S. Government Printing Office. https://www.oalj.dol.gov/LIBDOT.HTM

Veiersted KB, Forsman M, Hansson GA, Mathiassen SE (2013) Assessment of time patterns of activity and rest in full-shift recordings of trapezius muscle activity - effects of the data processing procedure. J Electromyogr Kinesiol 23(3):540-7 doi:10.1016/j.jelekin.2012.12.004

Veiersted KB, Westgaard RH, Andersen P (1993) Electromyographic evaluation of muscular work pattern as a predictor of trapezius myalgia. Scand J Work Environ Health 19(4):284-90

Villumsen M, Samani A, Jorgensen MB, Gupta N, Madeleine P, Holtermann A (2015) Are forward bending of the trunk and low back pain associated among Danish blue-collar workers? A cross-sectional field study based on objective measures. Ergonomics 58(2):246-58 doi:10.1080/00140139.2014.969783

Ware JE, Jr. (2000) SF-36 health survey update. Spine (Phila Pa 1976) 25(24):3130-9
